# Social Determinants of Health Associated With Unhealthy Weight Management Behaviors Among Emerging Adult Women: Nationwide Cross-Sectional Study

**DOI:** 10.2196/95474

**Published:** 2026-11-12

**Authors:** Jisu Lee, Hyeonkyeong Lee, Hyemi Sun, Chaehyeon Kang, Sun Young Shim

**Affiliations:** 1 Mo-Im Kim Nursing Research Institute, College of Nursing, Yonsei University, 50 Yonsei-ro, Seodaemun-gu, Seoul, Republic of Korea; 2 Institute for Innovation in Digital Healthcare, Yonsei University, Seoul, Republic of Korea

**Keywords:** unhealthy weight management behaviors, social determinants of health, emerging adults, body image, social norms, South Korea

## Abstract

**Background:**

Unhealthy weight management behaviors (UWMBs) have become increasingly common among emerging adult women, yet prior research has largely focused on psychological or perceptual correlates in isolation. In appearance-oriented contexts, structural influences, including social norms, may co-occur with individual characteristics to shape these behaviors. The social determinants of health (SDOH) framework offers a useful lens for examining UWMBs by jointly considering structural and intermediary determinants.

**Objective:**

This study aimed to identify key SDOH associated with engagement in UWMBs among emerging adult women in South Korea by examining structural and intermediary determinants within a single adjusted model.

**Methods:**

A nationwide cross-sectional survey was conducted among women aged 19 to 25 years using stratified sampling with quota allocation based on residential area and employment status. The final analytic sample included 1594 participants. UWMBs were assessed using a 9-item checklist; reporting at least 1 behavior in the past year was classified as UWMB engagement. Structural determinants included social norms, social influence, and socioeconomic position. Intermediary determinants included perceived body shape, body weight misperception, weight disparity, weight self-stigma, media health literacy, social network support, and BMI. Multivariable logistic regression analyses were performed to identify factors associated with UWMBs.

**Results:**

Overall, 88% (1404/1594) of participants reported at least 1 UWMB in the past year, and 71.4% (n=1002) engaged in 2 or more UWMBs. Compared with women without UWMBs, those engaging in UWMBs reported stronger weight-related social norms, greater social influence, more distorted perceptions of body image, higher weight self-stigma, and greater media health literacy and social network support. In the multivariable model, UWMB engagement was significantly associated with perceived body shape (average vs thin: OR 2.41, 95% CI 1.52-3.83; *P*<.001; obese vs thin: OR 2.33, 95% CI 1.13-4.79; *P*=.02), descriptive norms (OR 1.38, 95% CI 1.19-1.61; *P*<.001), thin-ideal internalization (OR 1.32, 95% CI 1.01-1.71; *P*=.04), BMI (OR 1.10, 95% CI 1.01-1.19; *P*=.04), and educational attainment (in college vs high school or less; OR 1.69, 95% CI 1.09-2.62; *P*=.02; college or higher vs high school or less; OR 1.81, 95% CI 1.13-2.91; *P*=.01), with perceived body shape showing the largest odds ratios.

**Conclusions:**

UWMBs are widely practiced among emerging adult women and are associated with both intermediary body image–related factors and broader sociocultural norms when considered simultaneously. These findings underscore the need for interventions that address appearance-related norms, particularly those disseminated through social media environments, rather than approaches focused solely on individual determinants. Future research should clarify the mechanisms linking structural and individual determinants to inform culturally relevant prevention strategies.

## Introduction

Heightened societal attention to appearance and weight stigma has intensified demand for weight loss [1], and unhealthy weight management behaviors (UWMBs) are increasingly adopted by emerging adult women [2]. These behaviors are associated with multiple adverse health outcomes, particularly eating disorders and psychological distress [3]. Social media and the growing availability of weight loss medications have further reinforced expectations of rapid and effortless weight reduction [4,5]. In the United States, more than half of emerging adult women have reported at least 1 UWMB, and more than 20% have engaged in extreme methods such as diet pills, self-induced vomiting, or the use of diuretics and laxatives [2]. As women at this life stage remain highly sensitive to appearance-related judgments [6] and prone to self-criticism [7], this pattern may extend beyond the United States and reflect a broader global trend. Similar concerns have also been reported in Asian [8] and European [9] populations. Moreover, longitudinal evidence indicates that UWMBs tend to emerge during emerging adulthood and may persist over time [2,8]. Therefore, identifying factors associated with UWMBs specific to emerging adult women is essential.

Distorted body image has been consistently identified as a key factor associated with UWMBs [10]. In particular, multiple forms of perceptual inaccuracy—such as heightened distortion in perceived body shape, discrepancies between actual and ideal body weight, and body dissatisfaction—are strongly associated with the adoption of unhealthy practices. These risks are particularly pronounced when thinness is internalized as the desirable body ideal. Various interventions targeting perceptual components of body image, including cognitive dissonance-based programs [11], self-compassion training [12], and media or health literacy education [13], have been developed to address this issue. However, such approaches largely remain centered on reshaping individual cognitions or attitudes and offer limited sustainability or responsiveness to broader sociocultural and structural forces that shape body image norms.

Beyond individual perceptions, social norms provide a structural context that reinforces weight concerns. Social norms refer to collectively shared expectations regarding acceptable or desirable body shapes within a given sociocultural environment. According to the tripartite influence model [14], family, peers, and media function as primary channels through which these appearance-related expectations are transmitted to individuals. This transmission operates through social influence, which encompasses the process by which socially shared norms are perceived as external pressure to conform to dominant body ideals and subsequently internalized as personal standards. With repeated exposure to such influences, body dissatisfaction and weight-related self-stigma intensify, and individuals’ body ideals progressively converge with prevailing sociocultural standards [15]. In South Korea, where thinness is culturally valued as a symbol of self-discipline and aesthetic capital [16], the prevalence of underweight remains high despite rising UWMBs [8], a pattern that may reflect the influence of social factors, particularly those conveyed through appearance-ideal content on social media [17]. Accordingly, media literacy, which enables critical evaluation of media messages, may serve as a potential protective factor [13].

Nevertheless, most prior research has examined factors associated with UWMBs in isolation, focusing on psychological or perceptual aspects such as body image, while broader structural determinants have received comparatively little attention. This fragmented perspective overlooks how social context and individual cognition interact to shape behavioral risk. To address this limitation, this study adopts the World Health Organization’s (WHO) social determinants of health (SDOH) framework [18], situating health behaviors within the interplay between structural determinants (eg, socioeconomic status [SES], social norms, and social influence) and intermediary determinants (eg, BMI, body image, and health literacy). Through this approach, the study aimed to identify SDOH empirically associated with UWMBs within the sociocultural context of emerging adult women.

## Methods

### Study Design

This cross-sectional study used primary data collected through a nationwide survey of emerging adult women aged 19 to 25 years in South Korea, referred to as the STOP 2025 survey. This age range reflects the conventional definition of emerging adulthood [19], excluding people aged 18 years, who are minors under Korean law. The study adhered to the STROBE (Strengthening the Reporting of Observational Studies in Epidemiology) guidelines for cross-sectional studies.

### Study Setting and Participants

The survey was conducted via an online panel managed by a professional survey research agency and drawn from the subscriber base of a major South Korean telecommunications provider. This panel included approximately 90,000 registered women aged 19 to 25 years, representing approximately 4.9% of the national female population in this age group. This limited coverage reflects the panel’s restriction to subscribers of a single telecommunications provider who had also enrolled as survey panelists.

Eligible participants were emerging adult women who could read and understand Korean and who provided informed consent prior to participation. Exclusion criteria, assessed via self-report at the online screening stage, included (1) severe cognitive impairment or communication difficulties, (2) current pharmacological treatment for a diagnosed eating disorder, or (3) medically supervised weight loss treatment for a chronic condition.

Stratified sampling with quota allocation was applied based on residential area (metropolitan vs nonmetropolitan) and employment status (employed vs nonemployed) to obtain a demographically diverse sample of women aged 19 to 25 years. Quota targets for these strata were set based on population statistics from Statistics Korea [20], approximating the actual distribution of women aged 19 to 25 years in South Korea (approximately equal proportions for both residential area and employment status).

The minimum required sample size was calculated using the Cochran formula for a single population proportion, assuming a prevalence of 30% [21], a 95% confidence level, and a 5% margin of error, yielding an initial estimate of 323 participants. We assumed that individuals within the same quota stratum might share lifestyle-related characteristics relevant to UWMBs (eg, consumption patterns and environmental exposure), potentially resulting in within-stratum correlation; accordingly, each quota stratum was conservatively treated as a cluster for the sample size calculation. In the absence of Korea-specific intraclass correlation coefficient (ICC) estimates for UWMBs, we applied an ICC of 0.039 [22] and a cluster size (m) of 100 (consistent with the Korea National Health and Nutrition Examination Survey [KNHANES] sampling convention), yielding a design effect of 4.861 and a required analytic sample size of approximately 1569. To allow for potential dropouts and exclusions, 1600 participants were recruited, allocated equally across the 4 resulting strata (400 participants per stratum), of whom 1594 were included in the final analysis. Six participants were excluded due to an outlier BMI value or logically inconsistent responses to the weight management behavior items.

### Measurements

#### Dependent Variable

UWMBs were assessed using a 9-item checklist adapted from Neumark-Sztainer et al [23]. Participants were asked, “During the past year, have you done any of the following to lose or maintain your weight?” Responses were recorded as yes or no for each of the following behaviors: (1) fasted, (2) ate very little food, (3) used food substitutes (eg, powders or diet drinks), (4) skipped meals, (5) smoked more cigarettes, (6) took diet pills (prescribed pills or diet-related health supplements), (7) induced vomiting, (8) used laxatives, and (9) used diuretics. Participants reporting at least 1 of these 9 behaviors were classified as having engaged in UWMBs.

#### Independent Variables

##### Structural Determinants

###### Social Norms

Two types of norms were assessed: descriptive norms (perceived prevalence of weight control behaviors among others) and injunctive norms (perceived expectations to engage in such behaviors). Both were adapted from Paek and Hove [24], with higher scores indicating stronger perceived norms. The norms are described as follows:

- Descriptive norms were measured with 2 items asking, “How many of the following people do you think are trying to stay thin or lose weight?” Responses were provided for (1) close friends and (2) same-age peers on a 6-point scale (1=“none” to 6=“80% or more”). The mean score across the 2 items was calculated (Cronbach α=0.813).
- Injunctive norms were measured with 3 items asking, “To what extent do the following people expect you to stay thin or lose weight?” Participants rated (1) close friends, (2) parents, and (3) significant others on a 5-point scale (1=“not at all” to 5=“very much”). The mean score across the 3 items was computed (Cronbach α=0.863).

###### Social Influence

Social influence, consisting of the internalization of appearance ideals and perceived appearance-related social pressure, was measured using the Korean version of the Sociocultural Attitudes Toward Appearance Questionnaire-4-Revised (SATAQ-4R) [25]. The instrument includes 31 items rated on a 5-point Likert scale (1=“strongly disagree” to 5=“strongly agree”), comprising 7 subscales assessing the internalization of thinness or low body fat, muscularity, and general attractiveness, as well as perceived pressure from family, peers, significant others, and media. Reverse-coded items were recoded prior to analysis. Higher scores on each subscale indicated stronger internalization or greater perceived pressure to conform to sociocultural appearance ideals. Cronbach α values ranged from 0.818 to 0.950 across subscales.

###### Socioeconomic Position

Socioeconomic variables included educational attainment, employment status, monthly income, marital status, household composition, residential area, and primary activity area. Subjective social status (SSS) was measured using the youth version of the MacArthur Scale of Subjective Social Status [26], with scores ranging from 1 (lowest) to 10 (highest).

### Intermediary Determinants

#### Perceived Body Shape

Perceived body shape was assessed using a single item from the KNHANES [21]: “What do you think about your body shape?” Responses were rated on a 5-point scale (1=“very thin” to 5=“very obese”) and were recoded into 3 categories by merging each extreme category with the adjacent category: thin (very thin and slightly thin), average, and obese (slightly obese and very obese).

#### Body Weight Misperception

Body weight misperception was defined as the discrepancy between actual BMI and perceived weight status. BMI was calculated from self-reported height and weight and classified according to Korean adult standards [27]: underweight (BMI <18.5 kg/m^2^), normal (BMI 18.5-22.9 kg/m^2^), and overweight (BMI ≥23.0 kg/m^2^). Perceived weight status was assessed using a single item [28]: “What do you think about your weight?” Responses were rated on a 5-point scale (1=“very underweight” to 5=“very overweight”) and were recoded into 3 categories corresponding to the BMI classifications described above. Body weight misperception was classified as accurate estimation (perceived weight category matching the BMI category), underestimation (overweight perceived as normal or underweight; normal weight perceived as underweight), and overestimation (underweight perceived as normal or overweight; normal weight perceived as overweight) [28].

#### Weight Disparity

Weight disparity was calculated as the ratio of desired weight to actual weight, expressed as a percentage. It was categorized into 3 groups: <100% (desired weight lower than actual weight), 100% (desired weight equal to actual weight), and >100% (desired weight higher than actual weight) [29].

#### Weight Self-Stigma

Weight self-stigma was measured using the Korean version of the Weight Self-Stigma Questionnaire (WSSQ) [30]. The 12 items were rated on a 5-point Likert scale (1=“completely disagree” to 5=“completely agree”), with higher scores indicating stronger internalized weight stigma (Cronbach α=0.914).

#### Media Health Literacy

Media health literacy was measured using a modified Korean version of the eHealth Literacy Scale (eHEALS) [31]. The 8 items were rated on a 5-point Likert scale (1=“strongly disagree” to 5=“strongly agree”), with higher scores reflecting a greater ability to find, evaluate, and use online health information (Cronbach α=0.914).

#### Social Network Support

Social network support was assessed using a modified Korean version of the Social Support for Exercise scale [32]. Although the cited scale’s items assessed perceived social support for exercise from family and friends, item wording in this study was modified to refer to support for weight control from people encountered on social media. The 6 items were rated on a 4-point Likert scale (1=“strongly disagree” to 4=“strongly agree”), with higher scores reflecting greater perceived support from social media–based networks for weight control (Cronbach α=0.943).

### Data Collection

Data were collected via an online self-administered questionnaire distributed by a professional survey agency over a 1-week period in January 2025. The research team provided the agency with the study information sheet, consent form, and questionnaire. Responses within each stratum were automatically closed to further submissions once the predefined quota was reached.

Survey invitations were sent to the provider’s nationwide panel via SMS and pop-up notifications. Participants accessed the questionnaire only after reviewing the online consent form and selecting “Agree.” Those selecting “Disagree” were automatically exited from the survey. Eligible respondents proceeded to a screening page, and only those meeting the inclusion criteria and none of the exclusion criteria completed the survey. Survey completion required approximately 30 minutes. All data were anonymized and deidentified by the survey agency prior to transfer to the research team.

### Statistical Analysis

Prior to analysis, the anonymized dataset was screened for normality assumptions, outliers, and missing data. Categorical variables were dummy coded, and negatively worded items were reverse scored. Descriptive statistics (means, SDs, frequencies, and percentages) were computed to summarize participants’ sociodemographic characteristics and the prevalence of UWMBs. No poststratification weighting was applied in the analysis. Group differences between women with and without UWMBs were first examined using independent *t* tests and chi-square tests, based on variables selected according to the SDOH framework. Subsequently, a multivariable logistic regression analysis was conducted by entering variables that were selected a priori based on the SDOH framework and that showed significant associations in bivariate analyses. Odds ratios (ORs) and 95% CIs were estimated from the multivariable logistic regression model. Multicollinearity among predictors in the multivariable model was assessed using variance inflation factors (VIFs). Model fit was evaluated using the Hosmer-Lemeshow goodness-of-fit test and Nagelkerke *R*^2^. Statistical significance was set at *P*<.05 (2-tailed). All analyses were performed using SPSS (version 29.0; IBM Corp).

### Ethical Considerations

This study was approved by the Institutional Review Board of Yonsei University Health System (4-2024-1456). A waiver of written consent was granted because the survey was conducted online through a professional research agency. Before participation, all respondents were presented with an online consent form describing the study purpose, procedures, confidentiality, and voluntary participation, including the right to withdraw at any time. Minimal personal information (sex, age, and residential area) was collected to verify eligibility and monitor quota compliance across strata. All responses were anonymized before being provided to the research team, and the survey agency permanently deleted all identifiable data after data delivery. Participants received a mobile gift voucher provided by the survey panel provider upon completion of the questionnaire. All procedures performed in this study were conducted in accordance with the ethical standards of the institutional review board and the Declaration of Helsinki and its later amendments.

## Results

### Participant Characteristics

The mean age of participants was 22.96 (SD 1.85) years. Most participants were currently enrolled in college (729/1594, 45.7%) or had graduated from college (n=539, 33.8%), and approximately half (n=796, 49.9%) were employed. The average SSS score was 5.26 (SD 1.64). The majority were unmarried (n=1537, 96.4%), and about one-quarter lived alone (n=402, 25.2%; Table 1).

**Table 1.** Socioeconomic position and sociodemographic characteristics of participants by unhealthy weight management behavior (UWMB) status among emerging adult women aged 19 to 25 years in a nationwide cross-sectional survey in South Korea, January 2025^a^.

Variables	Total (N=1594)	UWMB absent (n=190)	UWMB present (n=1404)	*χ*^2^ test (*df*)	*t* test (*df*)	*P* value
Age (years), mean (SD)	22.96 (1.85)	22.81 (1.88)	22.98 (1.84)		–1.20 (1592)	.23
Educational attainment, n (%)				9.9 (2)		*.*007
High school or less	326 (20.5)	54 (16.6)	272 (83.4)			
Enrolled in college	729 (45.7)	85 (11.7)	644 (88.3)			
College or higher	539 (33.8)	51 (9.5)	488 (90.5)			
Employment status, n (%)				6.0 (1)		*.*01
Unemployed	798 (50.1)	111 (13.9)	687 (86.1)			
Employed	796 (49.9)	79 (9.9)	717 (90.1)			
Monthly income (KRW)^b^, n (%)				12.5 (2)		*.*002
≤1 million	645 (40.5)	98 (15.2)	547 (84.8)			
1-2 million	344 (21.6)	39 (11.3)	305 (88.7)			
≥2 million	605 (38)	53 (8.8)	552 (91.2)			
Subjective socioeconomic status (range 1-10), mean (SD)	5.26 (1.64)	5.24 (1.73)	5.26 (1.63)		–0.17 (1592)	.87
Marital status, n (%)				6.7 (1)		*.*01
Unmarried	1537 (96.4)	177 (11.5)	1360 (88.5)			
Married	57 (3.6)	13 (22.8)	44 (77.2)			
Household composition, n (%)				2.0 (1)		.16
Single-person household	402 (25.2)	40 (10)	362 (90)			
Multiperson household	1192 (74.8)	150 (12.6)	1042 (87.4)			
Residential area, n (%)				0.4 (1)		.52
Metropolitan	798 (50.1)	91 (11.4)	707 (88.6)			
Nonmetropolitan	796 (49.9)	99 (12.4)	697 (87.6)			
Primary activity area, n (%)				0.4 (1)		.53
Metropolitan	814 (51.1)	93 (11.4)	721 (88.6)			
Nonmetropolitan	780 (48.9)	97 (12.4)	683 (87.6)			

a This table presents the socioeconomic position component of the structural determinants within the social determinants of health framework.

b 1 KRW=US $0.00075, as of October 28, 2026.

### Prevalence and Patterns of UWMBs

Among all participants (N=1594), 1404 (88%) reported engaging in at least 1 UWMB during the past year. The most frequently reported UWMB methods were eating very little food (n=1277, 80.1%) and skipping meals (n=720, 45.2%), followed by taking diet pills (n=545, 34.2%), using food substitutes (n=518, 32.5%), fasting (n=250, 15.7%), smoking more cigarettes (n=162, 10.2%), self-induced vomiting (n=90, 5.6%), laxative use (n=26, 1.6%), and diuretic use (n=23, 1.4%). Multiple UWMB methods were common: 1002 (71.4%) participants who engaged in UWMBs reported using 2 or more methods, whereas 402 (28.6%) reported using only 1 method.

### Group Differences by UWMB Status

As shown in Table 1, engagement in UWMBs differed significantly according to educational attainment, employment status, monthly income, and marital status. No significant differences were observed in age, SSS, household composition, or residential or primary activity area.

As shown in Table 2, significant differences between the UWMB and non-UWMB groups were observed in descriptive and injunctive norms and in sociocultural influences related to appearance, including internalization of appearance ideals and perceived appearance-related pressures. The groups also differed significantly in weight self-stigma, media health literacy, social network support, and BMI. Significant group differences were additionally found in perceived body shape and weight disparity, with a higher proportion of participants in the UWMB group reporting desired weights lower than their actual weights and perceiving themselves as obese. In contrast, body weight misperception did not differ significantly between the groups.

**Table 2.** Social norms, social influence, and intermediary determinants within the social determinants of health (SDOH) framework by unhealthy weight management behavior (UWMB) status among emerging adult women aged 19 to 25 years in a nationwide cross-sectional survey in South Korea, January 2025^a^.

Domains and variables	Total (N=1594)	UWMB absent (n=190)	UWMB present (n=1404)	*χ*^2^ test (*df*)	*t* test (*df*)	*P* value
Structural determinants						
Social norms, mean (SD)						
Descriptive norms	3.92 (1.20)	3.37 (1.29)	3.99 (1.17)		−6.76 (1592)	<.001
Injunctive norms	2.63 (1.07)	2.25 (1.06)	2.68 (1.06)		−5.25 (1592)	<.001
Social influence, mean (SD)						
Internalization: thin or low body fat	3.62 (0.90)	3.03 (0.93)	3.70 (0.87)		−9.83 (1592)	<.001
Internalization: muscularity	2.74 (0.88)	2.50 (0.79)	2.77 (0.89)		−3.99 (1592)	<.001
Internalization: general attractiveness	4.11 (0.67)	3.85 (0.70)	4.14 (0.65)		−5.83 (1592)	<.001
Pressures: family	2.33 (1.20)	1.88 (1.01)	2.39 (1.21)		−6.39 (268.128)	<.001
Pressures: peers	2.19 (1.07)	1.74 (0.88)	2.25 (1.08)		−7.39 (271.223)	<.001
Pressures: significant others	2.14 (1.13)	1.66 (0.87)	2.21 (1.15)		−7.71 (285.957)	<.001
Pressures: media	3.14 (1.27)	2.39 (1.19)	3.24 (1.24)		−8.84 (1592)	<.001
Intermediary determinants						
Perceived body shape, n (%)				121.8 (2)		<.001
Thin	308 (19.3)	92 (29.9)	216 (70)			
Average	689 (43.2)	65 (9.4)	624 (90.6)			
Obese	597 (37.5)	33 (5.5)	564 (94.5)			
Body weight misperception, n (%)				5.6 (2)		.06
Accurate estimation	1214 (76.2)	156 (12.9)	1058 (87.1)			
Underestimation	69 (4.3)	9 (13)	60 (87)			
Overestimation	311 (19.5)	25 (8)	286 (92)			
Weight disparity, n (%)				104.8 (2)		<.001
Desired weight=actual weight	104 (6.5)	20 (19.2)	84 (80.8)			
Desired weight<actual weight	1347 (84.5)	117 (8.7)	1230 (91.3)			
Desired weight>actual weight	143 (9)	53 (37.1)	90 (62.9)			
Weight self-stigma, mean (SD)	2.53 (0.84)	2.03 (0.76)	2.59 (0.83)		−8.79 (1592)	<.001
Media health literacy, mean (SD)	3.54 (0.68)	3.39 (0.76)	3.57 (0.66)		−3.05 (229.546)	.003
Social network support, mean (SD)	2.02 (0.83)	1.71 (0.77)	2.06 (0.83)		−5.46 (1592)	<.001
BMI (kg/m^2^), mean (SD)	21.74 (3.80)	19.83 (3.52)	22.00 (3.76)		−7.49 (1592)	<.001

a This table presents the social norms and social influence components of structural determinants within the SDOH framework, together with intermediary determinants.

### Factors Associated With UWMBs

Perceiving oneself as having an average or obese body shape, higher educational attainment, stronger descriptive norms, greater internalization of thinness or low body fat ideals, greater social network support, and higher BMI were significantly associated with increased odds of engaging in UWMBs (Table 3). No other factors remained significant after adjustment. VIF for all predictors in the multivariable model ranged from 1.03 to 4.26, indicating no substantial multicollinearity.

**Table 3.** Multivariable logistic regression of factors associated with unhealthy weight management behaviors (UWMBs) among emerging adult women aged 19 to 25 years in a nationwide cross-sectional survey in South Korea, January 2025 (N=1594)^a^.

Domains or variables	Odds ratio (95% CI)	*P* value
Structural determinants		
Social norms		
Descriptive norms	1.38 (1.19-1.61)	*<*.001
Injunctive norms	0.88 (0.73-1.07)	.20
Social influence		
Internalization: thin or low body fat	1.32 (1.01-1.71)	.04
Internalization: muscularity	1.13 (0.91-1.40)	.27
Internalization: general attractiveness	1.27 (0.94-1.73)	.12
Pressures: family	0.92 (0.73-1.15)	.44
Pressures: peers	0.89 (0.64-1.24)	.48
Pressures: significant others	1.18 (0.87-1.60)	.28
Pressures: media	1.11 (0.92-1.34)	.26
Educational attainment		
High school or less	1.00	—^b^
In college	1.69 (1.09-2.62)	.02
College or higher	1.81 (1.13-2.91)	*.*01
Employment status		
Unemployed	1.00	—
Employed	1.24 (0.77-2.01)	.38
Marital status		
Unmarried	1.00	—
Married	0.52 (0.24-1.10)	.09
Monthly income		
≤1 million	1.00	—
1-2 million	1.13 (0.70-1.81)	.61
≥2 million	1.71 (0.99-2.98)	.06
Intermediary determinants		
Perceived body shape		
Thin	1.00	—
Average	2.41 (1.52-3.83)	<.001
Obese	2.33 (1.13-4.79)	.02
Weight disparity		
Desired weight=actual weight	1.00	—
Desired weight<actual weight	1.02 (0.55-1.90)	.96
Desired weight>actual weight	0.78 (0.39-1.57)	.49
Weight self-stigma	1.28 (0.92-1.78)	.15
Media health literacy	1.17 (0.92-1.49)	.21
Social network support	1.29 (1.01-1.63)	.04
BMI (kg/m^2^)	1.10 (1.01-1.19)	.04

a Model fit: the adjusted model significantly improved model fit compared with the null model (*χ*2_23_=230.9; *P*<.001). The pseudo-*R*2 values were 0.135 for Cox and Snell and 0.260 for Nagelkerke. The adjusted model included all variables entered simultaneously into the regression model.

b Not applicable.

Participants who perceived themselves as having an average body shape showed the highest odds of UWMB engagement (OR 2.41, 95% CI 1.52-3.83), followed by those who perceived themselves as obese (OR 2.33, 95% CI 1.13-4.79), compared with those who perceived themselves as thin. Educational attainment showed a positive gradient: college students (OR 1.69, 95% CI 1.09-2.62) and college graduates (OR 1.81, 95% CI 1.13-2.91) had higher odds of engaging in UWMBs than high school graduates. Stronger descriptive norms (OR 1.38, 95% CI 1.19-1.61) and greater internalization of thinness or low body fat ideals (OR 1.32, 95% CI 1.01-1.71) were also significantly associated with UWMBs. Greater social network support (OR 1.29, 95% CI 1.01-1.63) and higher BMI (OR 1.10, 95% CI 1.01-1.19) were also positively associated with UWMB engagement.

## Discussion

### Key Findings

This study demonstrated that UWMBs were highly prevalent among emerging adult women in South Korea, underscoring their importance as a public health concern. Within the SDOH framework, this study found that perceived body shape emerged as the factor most strongly associated with UWMB engagement among emerging adult women in South Korea, supporting the existing body of knowledge. Notably, this study further highlights the influence of structural determinants—including descriptive norms and internalization of thin or low body fat ideals—on UWMBs. These findings extend prior research that has primarily emphasized individual psychological and perceptual correlates [10,15,16] by demonstrating that both intermediary and structural determinants remain salient when considered simultaneously. Therefore, UWMBs should be understood not as matters of personal choice but as behaviors embedded within social and structural environments, highlighting the need for structural approaches aimed at shifting sociocultural norms, alongside individual-level efforts.

The prevalence of UWMBs in this study (88%) was substantially higher than the 30% estimate in national statistics [21] and exceeded the international estimates reported in prior research (approximately 50%-60%) [2,23]. In this study, diet pills were operationalized to include weight loss supplements, consistent with a common definition of the term; however, as it is unclear whether diet pills were defined similarly in other studies, comparisons of prevalence estimates across studies should be made with caution. A persistent upward trend in behaviors such as meal skipping and diet pill use has also been observed in Korea over the past decade [8], suggesting a genuine increase in these behaviors over time within this population. Moreover, Korean women frequently attempt weight loss [33] and often engage in unhealthy methods [8], even when they have relatively low BMI or do not perceive themselves as overweight. Comparable patterns have been reported in other Asian countries, where young women show consistently high levels of weight loss attempts across the entire BMI distribution, substantially exceeding those observed in European, Mediterranean, and American populations [34]. Longitudinal studies from the United States have shown a marked increase in extreme weight control behaviors—a subset of the behaviors encompassed by UWMBs—from adolescence to adulthood [2], suggesting that similar upward trends may be occurring elsewhere. Taken together, these findings suggest that UWMBs may be increasingly normalized within the sociocultural context examined in this study, shaped by everyday appearance-based comparison in digital media [4], self-discipline–oriented views of weight control [16], and widely shared thinness norms [15]. Therefore, efforts to reduce or prevent UWMBs require careful consideration of both individual and structural contexts that may encourage emerging adult women to engage in these risky behaviors.

This study reaffirms prior evidence that distorted body image is a salient correlate of UWMBs [10], with perceived body shape emerging as the factor most strongly associated with UWMB engagement. Notably, even women who perceived themselves as having an “average” body shape showed an elevated likelihood of engaging in UWMBs. Previous studies have documented substantial discrepancies between objectively measured BMI and perceived body shape, even among individuals within the normal weight range, with some tending to perceive their body size as larger than it actually is [35]. Such perceptual discrepancies suggest that UWMBs may be more closely related to subjective, visually oriented standards of body evaluation than to objective weight status. This interpretation is consistent with contemporary shifts in body perception, particularly in the context of the expanding use of social media, where body evaluation increasingly relies on visual self-presentation and the perceived gaze of others rather than on measurable indicators such as BMI [36]. Overall, these findings indicate that perceived body shape reflects a socially mediated aspect of body image and call for more multidimensional approaches to its measurement. Unlike previous studies [10], weight disparity and body weight misperception were not significantly associated with UWMB engagement in the present study. Given the heterogeneity of body image indicators currently used in research, future assessment models should incorporate cognitive, emotional, perceptual, and sociocultural dimensions rather than relying on a single index or construct.

The specific role of social media in shaping perceived body shape remains exploratory, as social media use and appearance-related online behaviors were not directly assessed in this study. The perceived gaze of others may nonetheless be interpreted through the lens of objectification theory [37], which posits that women come to adopt an observer’s perspective on their bodies. This phenomenon has been consistently documented in social media–based appearance comparison research [4,36]. In the social media context, this has been conceptualized as appearance-related social media consciousness (ASMC), which shows substantial associations with body surveillance, body shame, and appearance comparison [38] and remains associated with disordered eating among emerging adults even after controlling for general social media use [39]. Other evidence suggests that exposure to appearance-ideal content, rather than social media use itself, is more strongly linked to body dissatisfaction [17]. Future research should clarify which of these mechanisms best accounts for the present findings.

The tripartite influence model [14] helps clarify why descriptive norms and internalization of thin or low body fat ideals emerged as significant structural factors associated with UWMBs in this study. Even though perceived pressures from peers, family, or media were not independently significant, repeated exposure to others’ weight control behaviors may normalize such practices and encourage similar engagement [40]; higher levels of social network support for weight control may likewise reflect embeddedness in appearance-oriented social environments in which dieting and weight control behaviors are more visible and socially reinforced, thereby amplifying descriptive norms. As these norms become more pervasive, appearance-related standards may be internalized as personal expectations even in the absence of explicit pressure. These mechanisms underscore the need for interventions that address both individual self-perception and the broader sociocultural norms that shape and reinforce it.

Unlike the SDOH perspective [18] and prior evidence suggesting that individuals with lower SES are more vulnerable to unhealthy behaviors [41], prior research indicates that women with higher SES tend to monitor their weight more strictly and attempt weight loss more frequently [42], and that even among underweight women, those with higher income levels report more frequent weight loss attempts [33]. In our study, monthly income, employment status, and marital status were associated with UWMB engagement in bivariate analyses, but only higher educational attainment remained significantly associated with greater odds of engaging in UWMBs after multivariable adjustment. These patterns imply that appearance-oriented cultural contexts [16] may create heightened vulnerability among women with higher educational attainment. Although higher educational attainment is associated with greater awareness of weight-related health information [43], which would generally be expected to reduce risky behaviors, media health literacy—the construct measured in this study—was higher among women engaging in UWMBs in the bivariate analysis but lost significance after adjustment. This pattern may reflect more active health information-seeking related to weight control, and may also indicate that eHEALS, as a unidimensional scale [31], does not capture the capacity to critically resist appearance-related media ideals. Future research should therefore operationalize SES in a manner that reflects the developmental characteristics of emerging adult women to further investigate these associations.

### Limitations and Future Directions

UWMBs were assessed using a self-reported behavioral checklist rather than a validated psychometric instrument and were operationalized as a broad binary outcome encompassing 9 behaviors of markedly different severities. This approach may have introduced measurement error and may have contributed in part to the high prevalence observed in this study. Given the resulting imbalance in the outcome distribution, the ORs are more appropriately interpreted as indicators of the direction and relative magnitude of associations rather than as precise estimates of relative risk. Similarly, the measure of social network support for weight control was modified from an existing exercise-related scale, and its validity for this purpose has not been established beyond internal consistency.

Separately, the survey response rate could not be obtained from the survey agency, which further limits the ability to assess nonresponse or self-selection bias in this online panel. Although quota targets were set to approximate the national population distribution [20], the panel was restricted to subscribers of a single telecommunications provider, and no poststratification weighting was applied. The sample should therefore not be assumed to be representative of the general population of South Korean emerging adult women.

A cross-sectional design precludes causal or directional inference, and because associations are situated within specific sociocultural and media environments, the generalizability of the findings to other populations, cultural contexts, or genders remains uncertain. Social media use and appearance-related online exposure were not directly measured, despite their plausible role in shaping UWMB engagement. In this context, although prior research has repeatedly validated pathways linking body image, body dissatisfaction, and disordered eating [4,44], such analyses have rarely been extended to UWMBs. To rigorously examine how these mechanisms operate within sociocultural contexts, future research should use structural modeling approaches, including mediation and moderation analyses.

Beyond theoretical refinement and in line with public health implications, future research should also focus on developing intervention strategies that mitigate the sociocultural norms constructed around the visible body. Social norm–based approaches have shown some promise in shaping health-related behaviors by altering perceived norms and collective expectations [45]. Likewise, media-based public campaigns that shift the focus from appearance to functionality or strength have demonstrated limited yet encouraging effects in improving body image outcomes [46]. Such interventions may gradually contribute to redefining appearance-centered values and promoting more inclusive, health-oriented social norms.

### Conclusions

This study demonstrates that UWMBs are highly prevalent among emerging adult women in South Korea and are shaped by both individual and structural determinants. UWMB engagement was associated not only with subjective body evaluation—particularly perceived body shape and BMI—but also with pervasive appearance-oriented social norms and the internalization of thin ideals. These cross-sectional findings suggest that UWMBs may not be effectively addressed through individual-level approaches alone, underscoring the potential need for interventions that target sociocultural norms while supporting healthier self-perceptions and more realistic body evaluations. Future research should clarify the pathways through which structural and individual determinants jointly relate to UWMBs using longitudinal designs and mediation analyses to inform culturally relevant prevention and intervention strategies. In addition, social media–based initiatives promoting diverse and health-oriented body ideals may represent an important strategy for reducing the social normalization of UWMBs.

## Abbreviations

ASMC: appearance-related social media consciousness
eHEALS: eHealth Literacy Scale
ICC: intraclass correlation coefficient
KNHANES: Korea National Health and Nutrition Examination Survey
OR: odds ratio
SATAQ-4R: Sociocultural Attitudes Toward Appearance Questionnaire-4-Revised
SDOH: social determinants of health
SES: socioeconomic status
SSS: subjective social status
STROBE: Strengthening the Reporting of Observational Studies in Epidemiology
UWMB: unhealthy weight management behavior
VIF: variance inflation factor
WHO: World Health Organization
WSSQ: Weight Self-Stigma Questionnaire
